# Release of eDNA by different life history stages and during spawning activities of laboratory-reared Japanese eels for interpretation of oceanic survey data

**DOI:** 10.1038/s41598-019-42641-9

**Published:** 2019-04-15

**Authors:** Aya Takeuchi, Takuya Iijima, Wataru Kakuzen, Shun Watanabe, Yoshiaki Yamada, Akihiro Okamura, Noriyuki Horie, Naomi Mikawa, Michael J. Miller, Takahito Kojima, Katsumi Tsukamoto

**Affiliations:** 1 Graduate School of Bioresource Sciences, Nihon University, 1866 Kameino, Fujisawa, Kanagawa 252-0880 Japan; 2 Department of Marine Science and Resources, Nihon University, 1866 Kameino, Fujisawa, Kanagawa 252-0880 Japan; 3 Department of Fisheries Faculty of Agriculture, Kindai University, 3327-204 Nakamachi, Nara, 631-8505 Japan; 4 IRAGO Institute, 377 Ehima-shinden, Tahara, Aichi 441-3605 Japan

## Abstract

To assist in detection of offshore spawning activities of the Japanese eel *Anguilla japonica* and facilitate interpretation of results of environmental DNA (eDNA) analysis in their spawning area, we examined the eDNA concentration released by each life history stage of artificially reared Japanese eels in the laboratory using quantitative real-time PCR (qPCR). We also compared eDNA concentrations between before and after artificially induced spawning activities. eDNA was not detected from three 30 L seawater tanks containing each single fertilized egg, but eDNA was found from other tanks each containing single individuals of larval stages (preleptocephalus and leptocephalus), juvenile stages (glass eel, elver and yellow eel) or adult stage (silver eel). The eDNA concentrations increased in the life history stages, showed a significant difference among all stages, and were positively correlated with the total length and wet weight. Moreover, the eDNA concentration after spawning was 10–200 times higher than that before spawning, which indicated that the spawning events in the ocean would produce relatively high eDNA concentration. These results in the laboratory suggested that eDNA analysis appears to be an effective method for assisting oceanic surveys to estimate the presence and spawning events of the Japanese eel in the spawning area.

## Introduction

The catadromous eels of the genus *Anguilla* are famous for their remarkable migrations between ocean and freshwater environments^1^. The Japanese eel is unique among the 19 anguillid eel species found throughout worldwide^2–4^ in being the only species whose spawning area have been determined by collecting their eggs, larvae and adults in the western North Pacific^5–8^. However, the natural spawning behavior of anguillid eels has never been observed, and many aspects of their spawning ecology are enigmatic.

The Japanese eel appears to spawn south of salinity fronts along the southern part of the West Mariana Ridge^7,9^ at new moon periods during the spawning season^10^. There remain questions such as about how they find mates to form spawning aggregations and how specific spawning sites are selected. Recent efforts have been made to further understand the spawning ecology of the Japanese eel through attempts to directly observe their natural spawning behavior using submersibles and underwater camera systems^11,12^. But, this requires that the precise location and depth of likely spawning are narrowed down to a specific area in advance for the underwater surveys. The inability to record the spawning behavior up to now is partly due to the vast scale of the spawning area (ca. 36,000 km^2^) and because the latitude of the spawning events can change among months and years depending on oceanographic conditions such as the location of the salinity front^7,9,13^. Other techniques are needed to determine where the eels may be aggregating before new moon periods, and then underwater surveys can be conducted where the eels are present during their spawning time.

Another technique has been used during recent oceanic surveys we conducted in the Japanese eel spawning area, which is to analyze environmental DNA (eDNA) from water samples to search for the presence of the adult eels. The water sampling, filtration, extraction and PCR system for the eDNA analysis worked successfully on board during an oceanic survey, and we could detect eDNA of the Japanese eel from 2 L seawater samples from water depths at 250 and 400 m using real-time PCR (not qPCR)^14^. This indicated that the onboard eDNA analysis was a viable technique for identifying locations or depths where Japanese eels may be within their spawning area during an oceanic survey.

Aquatic species release eDNA into water in the form of materials such as metabolic waste, feces, urine, mucus and gametes^15,16^. eDNA analysis can estimate species composition and distribution by confirming each unique sequence in environmental samples^17–19^. It has also been used to monitor spawning activities of fishes and amphibians in freshwater^20,21^. Detections of relatively high abundance of eDNA have shown that reproductive events occurred because adults released a large number of gametes during spawning^20,21^. Moreover, there were positive relationships between eDNA release rate and fish size/biomass^22–24^, so a high concentration of eDNA may locally occur around a site where adults aggregate. Therefore, it appears likely that offshore aggregations and spawning events of Japanese eels should be detectable by high eDNA concentrations.

Despite the widespread use of eDNA analysis in the field, less research has explored the nature of eDNA itself including its origin, state, transport and fate^16^. Interpretation of eDNA data must rely on inference because information such as the body size and sex of organisms cannot be obtained when using eDNA analysis^19^. This makes it unfeasible to infer the presence of Japanese eels or the occurrence of their spawning activities during eDNA oceanic surveys. Our detection of Japanese eel eDNA in the spawning area indicated that eels could be present near the water sampling sites but it did not provide evidence of the spawning activities^14^. Not enough is known yet about the nature of eDNA to be able to infer that spawning events have occurred, such as how long does the eDNA persist, how much does the eDNA concentration change between before and after spawning, and what is the effect of life history stages on eDNA concentrations. The eDNA release rate was 3–4 times higher in adults than in juveniles of bluegill sunfish^24^ and might be determined by animal sizes^25,26^. Therefore, both the life history stages with different body sizes and the occurrence of spawning activities would affect the eDNA amount released into the water before a water sampling. However, the effect of these factors on eDNA concentration in water has not been studied for the Japanese eel.

To facilitate to infer the presence of different life history stages and the occurrence of spawning activities using eDNA analysis, we examined 1) the eDNA concentration released by each life history stage of laboratory-reared Japanese eels: egg, larval stages (preleptocephalus and leptocephalus), juvenile stages (glass eel, elver and yellow eel) and adult stage (silver eel), and 2) the change of the eDNA concentration between before and after artificially induced spawning. This new information should be useful in interpretation of eDNA that would be detected in the spawning area of the Japanese eel.

## Methods

Two types of experiments were conducted from November to December 2016 to examine the effect of 1) different life history stages, and 2) the spawning activities of the Japanese eel, on eDNA concentration. The experiments were conducted at the IRAGO Institute (Aichi, Japan), which is located in a coastal area, and seawater (ca. 32.9–33.9 psu) from saline groundwater was used. Three 500 mL seawater samples were analyzed to confirm the absence of eDNA of the Japanese eel in the seawater using the quantitative real-time PCR (qPCR) prior to the two experiments. All equipment (e.g. tanks, hoses and containers) were washed with or dipped in a 0.5% bleach solution for at least 5 min to remove any eDNA and then rinsed with the seawater before being reused. The filtration, eDNA extraction and qPCR set-up process were conducted in separate working places where PCR products have not been handled, and a qPCR machine was set outside of those places. All working places were sterilized, filter pipet tips were used, and disposable gloves were changed between each sample and process to avoid contamination.

### Water sampling

**Figure 1:**
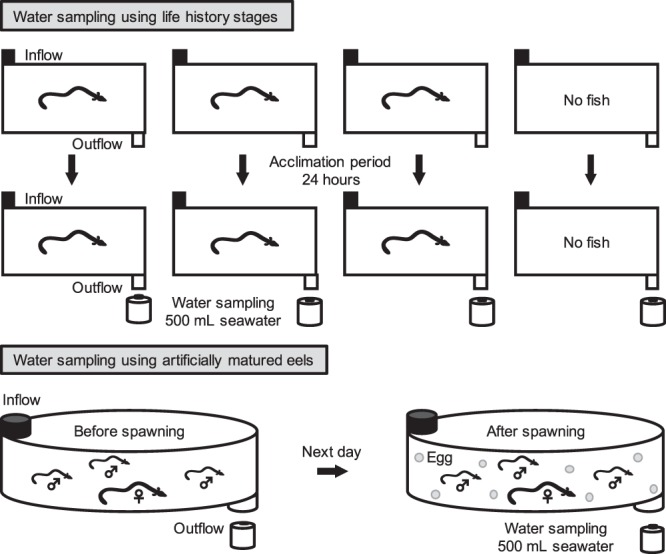
Experimental design showing the two types of tanks used to hold the different life history stages of the Japanese eel (top) and the artificially matured eels (bottom). Seawater was sampled from each outflow for eDNA analyses. Three tanks with single individuals and a negative control tank (all 30 L) were set-up seven times (each stage) for testing the different stages. Two polyvinyl tanks with a capacity of 1000 L seawater were prepared for holding one artificially matured female eel and three males that spawned in each tank.

To quantify the eDNA concentration in surrounding water released by each stage, three laboratory-reared individual eggs, preleptocephali, leptocephali, glass eels, elvers, yellow eels and silver eels were used and tested individually in three separate tanks at the same time for each stage. All silver eels used in this study were female because male silver eels may have already begun ejaculation which might show high concentration of eDNA (see Results). We measured egg diameter, total length and wet weight of each given individual before the water sampling (Table 1). The wet weight per individual of the eggs and preleptocephali were estimated by dividing the total wet weight by the number of individuals examined. No food had been given to all eels for at least 2 days before each water sampling to reduce the effect of their feces on the eDNA concentration as possible. Four 30 L flow-through tanks (38.5 × 50 × 30 cm) were prepared for testing each stage at separate times. The water temperatures and the flow through rates were 24.3–25.1 °C and 0.7–1.03 L min^−1^ during the experiment, respectively. A single individual of each developmental stage was introduced into each of the three tanks, and the forth tank without fish served as a tank blank during each trial for the different seven stages (Fig. 1). Because eggs would have residual eDNA from adult eels after artificially induced spawning, each egg was introduced into a tank after being carefully rinsed with the seawater. A 24-hour acclimation period was set to allow the extraordinary activity of the larvae or eels to decline after they were introduced into the tanks^24^. 500 mL of seawater was collected from the outlet of each of the four tanks using a disposable plastic bag (Yanagi, Aichi, Japan) after the acclimation period (Fig. 1). We eventually obtained 21 seawater samples and seven tank blanks in this experiment.

**Table 1:** Total lengths and wet weights of all life history stages of the Japanese eel.

Life history stage	Age	Total length (mm)	Wet weight (g)	Mean eDNA (copies µL^−1^)
Egg	−1.25 dph	1.12 (diameter)	0.0013^a^	0
	−1.25 dph	1.12 (diameter)		0
	−1.25 dph	1.49 (diameter)		0
Preleptocephalus	0.1 dph	3.54	0.00022^a^	0
	0.1 dph	4.27		0.2^b^
	0.1 dph	4.04		0.1^b^
Leptocephalus	211 dph	41	0.17	1.4
	211 dph	36	0.18	2.9
	211 dph	32	0.1	2.4
Glass eel	219 dph	47	0.13	0.4
	219 dph	49	0.12	0.3
	219 dph	48	0.12	0.5
Elver	ca. 7 mths	129	1.67	9.4
	ca. 7 mths	122	1.36	7.6
	ca. 7 mths	114	1.12	6.1
Yellow eel	ca. 1.5 yrs	498	159	6.7
	ca. 1.5 yrs	524	167	133.1
	ca. 1.5 yrs	445	134	184.8
Silver female eel	ca. 2.5 yrs	770	500	293.7
	ca. 2.5 yrs	640	390	206.3
	ca. 2.5 yrs	650	280	446.9

0 copies µL^−1^ shows that no eDNA was detected in three qPCR replicates. dph, days post-hatching; mths, months; yrs, years. ^a^Values divided the total wet weight by number of individuals examined. ^b^Samples showing one eDNA amplification in three qPCR replicates.

To test the effect of spawning activities on eDNA concentration, two artificially matured female eels and six artificially matured male eels were used. They were anesthetized for measurement of their total lengths and wet weights before the experiment (Table 2). There was no eDNA originating from the feces because the eels remained unfed during artificial induction of maturation for about two months or more. Two flow-through tanks with a capacity of 1000 L (Tank 1 and Tank 2) were prepared. A net with less 0.5 mm mesh size was set in each outlet of the two tanks to prevent eggs from flowing out. The water temperatures and flow through rates were 22.6–23.6 °C and 3.0–3.2 L min^−1^ during the experiment, respectively. After the two tanks were filled with seawater, a 500 mL seawater sample was collected from each outlet of the tanks using the disposable plastic bag and served as a tank blank. The eels were given a final hormonal treatment to induce artificial spawning^27^, and one artificially matured female eel and three artificially matured male eels were immediately put in each tank at about 17:00. 500 mL of seawater was collected before spawning using the disposable plastic bag from each tank at about 19:00 (Fig. 1). The eels probably spawned from midnight to early morning, and then 500 mL of seawater was collected at 8:00 of the next day when eggs were observed in the tanks. We eventually obtained four seawater samples and two tank blanks in this experiment.

**Table 2:** Total lengths and wet weights of two artificially matured female and six male eels.

Sex	Tank 1		Tank 2	
	Total length (mm)	Wet weight (g)	Total length (mm)	Wet weight (g)
Female	810	1409	800	1060
Male	590	322	650	338
	710	530	630	364
	738	592	731	608

### Filtration, extraction and quantitative real-time PCR

All water samples and tank blanks were stored in an ice-filled container and transported from the institute to another laboratory within 20 minutes soon after each water sampling for each of the eight experiments (seven stages and the spawning experiment). All transported water samples and tank blanks were quickly filtered using filter cartridges (pore size, 0.45 µm; Merck Millipore, Darmstadt, Germany). 500 mL of pure water was filtered after the filtration of all seawater samples collected each day, which produced nine filtration blanks. Soon after filtration, eDNA was extracted from the filter cartridges using DNeasy Blood and Tissue Kits (Qiagen, Hilden, Germany) in a final volume of 100 µL according to the manufacturer’s instruction and Miya *et al*.^28^. Nine extraction blanks were processed in parallel with seawater samples to monitor possible contamination. All eDNA extracts including the tank samples, filtration blanks and extraction blanks were stored −20 °C for a subsequent qPCR analysis.

The qPCR was performed using a Light Cycler® Nano (Roche, Basel, Switzerland). Primers and a probe (Table 3) were previously designed to be specific to the Japanese eel and produced a 154 bp amplicon within the mitochondrial 16S rRNA gene^29,30^. The specificity of the primers and probe for the Japanese eel have been confirmed using tissue-derived DNA extractions of 3 *Anguilla* (*A. japonica*, *A. marmorata* and *A. bicolor pacifica*) and 6 other anguilliform species (*Conger myriaster*, *Strophidon ui*, *Rhinomuraena quaesita*, *Uropterygius* sp., *Stemonidium hypomelas* and *Serrivomer sector*)^29,30^. These species are closely related to the Japanese eel and could be present around the spawning area of the Japanese eel. The same primers and probe also enabled us to specifically detect eDNA of the Japanese eel from a tank and the open ocean^14^. Each 10 µL qPCR reaction consisted of 500 nM of each primer, 1.9 µL of H_2_O PCR grade (Roche), 100 nM of a hydrolysis probe, 5 µL of 2 × Master Mix (Roche) and 2 µL of an eDNA extract as a template. The qPCR condition consisted of an initial activation step of 600 s at 95 °C, and 55 two step cycles of 20 s at 95 °C and 40 s at 60 °C. All eDNA extracts were amplified in triplicate 10 µL qPCR solutions, and each qPCR run included one PCR blank. For each qPCR run, a six-point standard curve with three replicates for plasmid DNA at known concentrations (3 × 10^0^–3 × 10^5^ copies µL^−1^) was used to estimate absolute eDNA concentration in seawater samples. In all qPCR runs, the standard curve slopes ranged from −3.83 to −3.62, y intercepts ranged from 38.4 to 39.3 cycles, R^2^ values ranged from 0.9975 to 1, and efficiencies ranged from 82.4 to 88.9% (Supplementary Table S1). Because 0.1 copy µL^−1^ of the eDNA from a preleptocephalus was detected in at least one of three replicates (see Results), we defined the limit of detection for the qPCR system as 0.1 copy µL^−1^. Amplicon presence was verified by agarose gel electrophoresis using a 2% agarose gel, stained with SYBR® safe DNA gel stain (Invitrogen, Japan, Tokyo) and visualized under ultraviolet light on Gel Doc^TM^ EZ imager (Bio Rad, CA, USA). We considered a sample showing ≥0.1 copy µL^−1^ and the amplicon presence to be positive.

**Table 3:** Specific primers and probe for the Japanese eel that target 154 base pairs of the mitochondrial 16S rRNA gene.

Primer and probe	Sequences (5′-3′)
Forward primer	AATCAGTAATAAGAGGGCCCAAGC
Reverse primer	TGTTGGGTTAACGGTTTGTGGTA
Probe	CACATGTGTAAGTCAGAACGGACCGACC

### Data analysis

Mean eDNA concentration (copies µL^−1^) of each life history stage was calculated using each of three measured values per a water sample except for eggs (no detections) and preleptocephali (partial detections) (Table 1). When no eDNA was detected in three qPCR replicates, the mean eDNA was considered as 0 copies. Single measured values of eDNA from two preleptocephali were directly used for data analysis. A Kruskal-Wallis test was used to examine differences in mean eDNA concentration among all developmental stages. Pairwise tests between preleptocephali and leptocephali, leptocephali and glass eels, glass eels and elvers, elvers and yellow eels, and yellow eels and silver eels were done with a Welch’s t-test. A general linear model (GLM) was used to determine whether the eDNA concentration was related to the total length and wet weight of the stages. Except for eggs, each mean eDNA value of preleptocephali, leptocephali, glass eels, elvers, yellow eels and silver eels was used for the GLM (*n* = 18). All statistical analyses were conducted in R ver. 3.3.2. eDNA concentration per wet weight (copies g^−1^) was calculated by dividing each mean eDNA concentration by each wet weight of preleptocephali, leptocephali, glass eels, elvers, yellow eels and silver eels.

### Ethical statement

The experiments were conducted in accordance with the relevant guidelines and regulations, and our protocols were approved by Institutional guidelines for animal experiments of Nihon University.

## Results

### eDNA release of each life history stage

**Figure 2:**
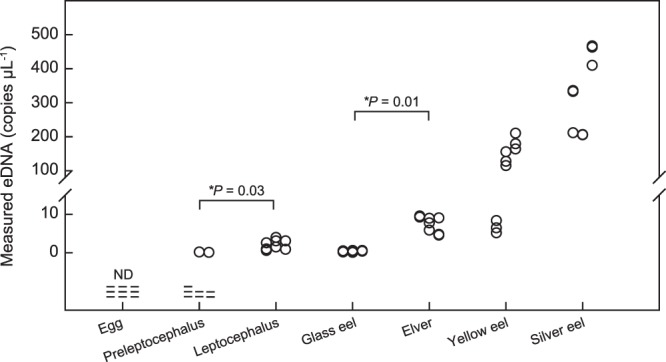
eDNA concentration (copies µL^−1^) released by each life history stage and statistical comparison results. Dashes show non-detections of eDNA (ND).

The eDNA of the Japanese eel was detected from tanks that contained a single individual of a preleptocephalus, leptocephalus, glass eel, elver, yellow eel and silver eel, while no eDNA was detected from tanks that contained a fertilized egg (Fig. 2, Supplementary Table S2). The eDNA from a preleptocephalus was detected from two of the nine qPCR solutions but not the seven other replicates. Therefore, when a single preleptocephalus was introduced into the 30 L seawater tank, false negatives (eDNA is not detected where the target species is present) occurred at about 78% probability. The measured values of detected eDNA were 0.1 and 0.2 copies µL^−1^ for each preleptocephalus, 0.6–4.0 copies µL^−1^ for each leptocephalus, 0.1–0.6 copies µL^−1^ for each glass eel, 4.6–9.6 copies µL^−1^ for each elver, 5.2–210.6 copies µL^−1^ for each yellow eel, 205.8–467.2 copies µL^−1^ for each silver eel (Fig. 2). This showed that eDNA concentration tended to be higher as the life history stages progressed (Fig. 2). The size difference was small between the leptocephali (36 mm in mean TL, Table 1) and the slightly larger glass eels (48 mm), but the eDNA concentration of the leptocephali (2.2 copies µL in mean concentration) was slightly higher than glass eels (0.4 copies µL). There was a statistically significant difference in the mean eDNA concentration (copies µL^−1^) among all life history stages (Kruskal-Wallis test, *P* < 0.05). The mean eDNA concentration was significantly different between preleptocephali and leptocephali (*P* = 0.03), glass eels and elvers (*P* = 0.01) (Fig. 2, Welch’s t-test). In contrast, there was no statistically significant difference in the mean eDNA concentration between leptocephali and glass eels (*P* = 0.05), elvers and yellow eels (*P* = 0.19), yellow eels and silver eels (*P* = 0.08) (Fig. 2, Welch’s t-test). One out of three yellow eels examined showed 5–10 copies µL^−1^ at low eDNA concentration which was similar to the same level with the eDNA concentration of elvers (Table 1, Fig. 2). No abnormal behavior was observed in any of the eel stages during the experiment. All blanks turned out negative, showing no indication of contamination.

**Figure 3:**
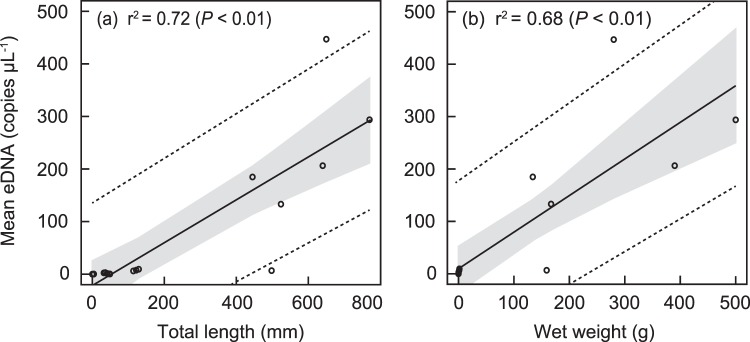
Relationship between mean eDNA concentration and total length (a), and wet weight (b) using GLM for 18 data. Solid lines, gray shadows, dotted lines and r^2^ show regression lines, 95% confidence limits of regression lines, 95% prediction limits of mean eDNA concentration data and coefficients of determination, respectively.

**Figure 4:**
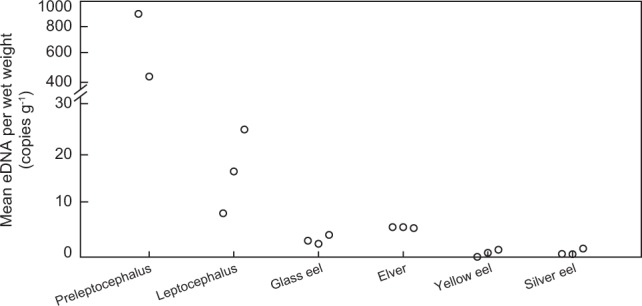
Mean eDNA concentration per wet weight (copies g^−1^). No eDNA was found from one preleptocephalus (See Table 1).

The general linear model revealed a positive correlation between the total lengths and the mean eDNA concentration released by preleptocephali, leptocephali, glass eels, elvers, yellow eels and silver eels (the coefficient of determination: r^2^ = 0.72) (*P* < 0.01, Fig. 3a). There was also a positive correlation between the wet weights and the mean eDNA concentration of each stage (r^2^ = 0.68) (*P* < 0.01, Fig. 3b). The mean eDNA concentration of the above-mentioned stages increased as the total lengths and wet weights were larger (Table 1, Fig. 3). The eDNA concentration per wet weight (copies g^−1^) of a preleptocephalus was much higher compared with yellow eels and silver eels, and the ratio decreased as the stages progressed (Fig. 4). Contrarily, the eDNA concentration per total length (copies mm^−1^) increased as the stages progressed (Supplementary Fig. S1).

### eDNA concentration before and after spawning

**Figure 5:**
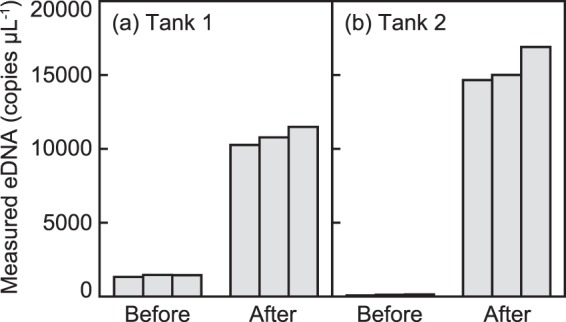
eDNA concentration (copies µL^−1^ in three replicates) before and after spawning in two tanks.

The eDNA concentration of the three replicates of Tank 1 were 1328, 1460 and 1443 copies µL^−1^ before spawning, and 10,263, 10,777 and 11,484 copies µL^−1^ after spawning (Fig. 5a). Those of Tank 2 were 70, 124 and 139 copies µL^−1^ before spawning, and 14,655, 15,003 and 16,892 copies µL^−1^ after spawning (Fig. 5b). The eDNA concentrations after spawning were approximately 10–200 times higher than those before spawning. After spawning, we observed the eggs, small pieces of the ovarian tissue and distinct milky water including the sperm in the tanks, all of which were considered as eDNA sources from the artificially matured adult eels. All blanks showed no DNA amplification, and therefore no contamination occurred during the experiment.

## Discussion

### eDNA release by different life history stages

The eDNA release from Japanese eels increased as the life history stages progressed, which could be attributed to the growth of the body size such as the total length and wet weight. This result corresponded with the hypothesis that eDNA production may depend on animal size^25,26^. As the body size enlarges, the skin area in contact with aquatic environments is increased, which would contribute to the increase of eDNA concentration in the water. Upon death or injury epithelial cells in fish peel off and are replaced with new cells from the basement membrane to maintain the tissue^31^. The external surface of the body of the Japanese eel has skin mucus that provides resistance to infectious diseases, and pathogens invading into the skin can be released together with mucus including old cells into the surrounding water^32^. Bigger eels likely release larger amounts of sloughed cells, mucus and some materials containing DNA.

The total length and the wet weight are not the only factors to consider though, because the body surface area of animals may be an important factor affecting the eDNA production. The mean eDNA concentration and the wet weight of leptocephali were slightly higher than those of glass eels even though glass eels were slightly larger in total length (Table 1). The body depth and total length of leptocephali decrease after they metamorphose into glass eels, and the surface area also decreases by one-third from 8.0 to 2.3 cm^2^ ^33,34^. Thus, the slightly higher eDNA concentration for leptocephali may have resulted from the difference of body size including surface area.

Another finding was that the eDNA of individual fertilized egg was not detected in the 500 mL samples from the 30 L flow-through tanks. The egg envelope of teleost fishes consists of 3–4 proteins that maybe involved in the hardening of the egg envelope after fertilization^35^. When the Japanese eel hatches out, the egg envelope is digested by proteolytic hatching enzymes secreted from hatching gland cells of the embryo^36^. After eggs are fertilized, nuclei and mitochondria including DNA are likely isolated from surrounding environments by the hard and acellular egg envelope. The egg envelope of fishes may allow selective transport of water and nutrients into a developing oocyte^37,38^, while possibly acting as a barrier for movement of high molecular weight organic compounds^39^ such as DNA. Although DNA transport from the embryo to surrounding water is uncertain, the eDNA release from an egg must have been below the limit of detection for the qPCR system even if the eDNA could pass through the egg envelope and come out into the water. There is also a possibility that the eggs never release any genetic materials, so future studies using a large number of eggs need to be conducted.

Interestingly, the eDNA concentration per wet weight (copies g^−1^) declined as the life history stages grew (Fig. 4). This was especially clear for the small preleptocephali, which had disproportionally much higher levels of eDNA release compared to their weight than the other stages. In another study, the eDNA release rate per wet weight (copies h^−1^) was slightly higher in the juvenile group of bluegill sunfish than in the adults, which could be because of an ontogenetic decrease in the metabolic rate^24^. The metabolic rate also decreased with increasing body mass in carp, porgy and four species of leptocephali^40,41^. These studies indicated that the decline of the metabolic rate per the body mass resulted from a combination of an increase in the relative size of tissues of low metabolic activity and a decrease in the metabolic activity of tissues with increasing body mass. The eel larvae actively metabolized and excreted large amounts of metabolic waste compared with their small wet weight because they required more combustible energy to quickly increase the body size^41^. The decrease in eDNA concentration per wet weight might be explained by larvae having a higher metabolic rate per gram compared to adult fish. This possibility might be examined by comparison of eDNA concentration released by an adult and by many larvae with the same total weight as the adult. A high eDNA concentration should be detected from a tank containing many larvae.

The eDNA release may also differ among some individuals of the same developmental stage. In the present study, one yellow eel had a low eDNA concentration of 6.7 copies µL^−1^ compared to the other two individuals (ca. 130 copies µL^−1^) (Table 1, Fig. 2a). It is unclear which of these two levels of eDNA concentration is normal, but because the low eDNA concentration of one yellow eel was identical with that of the three elvers with much smaller body size, it seems that the one yellow eel produced a unusually low concentration of eDNA or there was some sort of bias in eDNA distribution within the tank at the time of sampling. The individual variability in eDNA release may be due to differences in the stress and activities of the eels in each tank although their behavior appeared to be similar in this study. Individual differences were also found in the eDNA released by salamanders^26^, but the cause of individual differences has never been examined. Further studies addressing what mechanisms influence eDNA origin and production would improve the understanding of how eDNA detection is related to the presence of a species.

### eDNA release during spawning

The eDNA detected after spawning was 10–200 times higher than that before spawning in the two tanks each containing one artificially matured female eel and three males (Fig. 5). This clearly suggested that huge amounts of eDNA were released by the spawning activities. The result is previously expected because gametes would be eDNA sources^17,20^, and we collected seawater samples from the tanks containing the eels the day after spawning that occurred during nighttime. Strong eDNA signals have been found to reflect the spawning events of the macquarie perch *Macquaria australasica* and eastern hellbender *Cryptobranchus alleganiensis alleganiensis* that use external fertilization in freshwater areas^20,21^. Our finding of greatly increasing eDNA concentration in the tank after spawning supports the inference that a distinct change of eDNA concentration can result from spawning events in water, including by the Japanese eel in the offshore spawning area.

The huge amounts of eDNA detected after spawning in the laboratory most likely originated from the sperm of matured males. The sperm of the Japanese eel, whose head length and head width are 5 µm and 1 µm respectively, has a single mitochondrion^42^. The pore size of the filters used in this study was 0.45 µm, which was sufficient for collecting the sperm. Other genetic materials are likely contained in ovarian fluid and tissues from the females, as well as the skin mucus of the artificially matured Japanese eels in the tanks.

### Detection of Japanese eel spawning events in the ocean

To be able to interpret any detections of Japanese eel eDNA in their spawning area, we must consider various factors. The present study provided some crucial information about the eDNA concentration released by each life history stage and during spawning events. The experiment showed that different stages produced different concentrations of eDNA, but all of the concentration will be diluted over time by water mixing within the current and eddies of the North Equatorial Current that passes through the spawning area of the Japanese eel^13,43^. Another important factor is the spawning biology of the Japanese eel that spawns 2–4 days before new moon during each month of their spawning season^7,10^. Because of this, any eDNA found in the West Mariana Ridge spawning area before each new moon is most likely from silver eels preparing for spawning, since the larvae from the previous month’s spawning would have been transported out of the area. Any eDNA detections during oceanic surveys might show lower concentrations (after dilution and degradation) than the eDNA concentrations of the silver eels and of the spawning activities tested in the present study.

After spawning in the ocean, the eDNA detections could come from the preleptocephali, leptocephali or silver eels. Our two experiments showed that the eDNA concentration from silver eels was about 70–4000 times higher than those from the larvae in 30 L seawater tanks, and that the spawning activities produced high eDNA concentrations. This indicates that if a relatively high eDNA concentration was detected in the spawning area, the eDNA would most likely originate from the spawning events of the adult eels.

Other factors can also be used to interpret eDNA detection in the spawning area, such as the depth where positive samples were collected. For example, eDNA from the Japanese jack mackerel *Trachurus japonicus* and Japanese sea nettle *Chrysaora pacifica* were detected from surface water and sea bottom water where each species was mainly distributed, respectively^44,45^. The exact spawning depth of the Japanese eel is not known, but collections of their eggs and newly hatched preleptocephali were concentrated at depths of about 160–180 m^7,9^, suggesting that the eels spawn at or below those depths. Another factor is that migrating Japanese eels monitored by satellite transmitting pop-up tags found that they swam at about 180–250 m water depths at night and at about 500–900 m in the daytime^46,47^. This indicated that eDNA detection in the upper 200 m may be from the preleptocephali, leptocephali or possibly silver eels, while the detection deeper than 200 m would be from silver eels.

The eDNA concentration released from Japanese eels, degradation and dilution with distance from release, and depth and time of eDNA detection all need to be factored into the water sampling design and interpretation of results of oceanic surveys in the spawning area. Our detection of Japanese eel eDNA during a previous survey using a real-time PCR system (not qPCR) after the spawning had likely finished was difficult to interpret^14^. However, the deep depths of the detections (200–400 m) suggested that the eDNA was likely from adults and not from larvae since preleptocephali can be found in the shallower waters less than 200 m. Our findings about eDNA concentration from each life history stage of the Japanese eel and from spawning activities provide valuable criteria to assist in evaluating quantitative data of eDNA that could be obtained from oceanic surveys. The criteria can be used with samples analyzed before spawning to locate areas where eels may spawn (presence of pre-spawning silver eels), during spawning (evaluate timing and depths), and then after spawning to determine where multiple spawning events may have occurred. Therefore, the eDNA analysis appears to be a promising approach for assisting oceanic surveys using submersibles and underwater camera systems to attempt to observe natural spawning behavior of the Japanese eel. This analysis can also be transferable to estimate other anguillid spawning areas, which would help in understanding the spawning ecology of these mysterious fishes that spawn offshore in the ocean.

## Supplementary information

Supplementary Information

## Data Availability

All data generated during this study are available in the Supporting Information file.
