# Supplementary material for: Release of eDNA by different life history stages and during spawning activities of laboratory-reared Japanese eels for interpretation of oceanic survey data
Source: Sci Rep. 2019 Apr 15;9:6074. doi: 10.1038/s41598-019-42641-9 (PMC6465351; doi:10.1038/s41598-019-42641-9)
Supplement: Supplementary file 1 — Supplementary Information [file 41598_2019_42641_MOESM1_ESM.docx]

**Supplementary Information**

**Release of eDNA by different life history stages and during spawning activities of laboratory-reared Japanese eels for interpretation of oceanic survey data**

Aya Takeuchi^1,*^, Takuya Iijima^1^, Wataru Kakuzen^2^, Shun Watanabe^3^, Yoshiaki Yamada^4^, Akihiro Okamura^4^, Noriyuki Horie^4^, Naomi Mikawa^4^, Michael J. Miller^2^, Takahito Kojima^1,2^ and Katsumi Tsukamoto^1,2^

^1^Graduate School of Bioresource Sciences, Nihon University, 1866 Kameino, Fujisawa City, Kanagawa 252-0880, Japan; ^2^Department of Marine Science and Resources, Nihon University, 1866 Kameino, Fujisawa City, Kanagawa 252-0880, Japan; ^3^Department of Fisheries Faculty of Agriculture, Kindai University, 3327-204 Nakamachi, Nara 631-8505, Japan; ^4^IRAGO Institute, 377 Ehima-shinden, Tahara, Aichi 441-3605, Japan

*Corresponding author: Aya Takeuchi

Address: Graduate School of Bioresource Sciences, Nihon University, 1866 Kameino, Fujisawa City, Kanagawa 252-0880, Japan

Email: ka030ak@yahoo.co.jp

**Contents**

**Figure S1.** Mean eDNA concentration per total length (copies mm^-1^).

**Table S1.** Results of all qPCR analysis.

**Table S2.** Measured eDNA concentration of each life history stage of artificially reared Japanese eels.

**Figure S1.** Mean eDNA concentration per total length (copies mm^-1^). No eDNA was found from one preleptocephalus (see Table S2).

**Table S1.** Results of all qPCR analysis. 12 qPCR runs were performed for the eDNA analysis of two experiments (testing the effect of different life history stages and artificially induced spawning on eDNA concentration).

| qPCR run | Slope | Y intercept | R^2^ | PCR efficiency |
| --- | --- | --- | --- | --- |
| # 1 | -3.80 | 39.04 | 0.9999 | 83.3 |
| # 2 | -3.70 | 38.87 | 0.9988 | 86.3 |
| # 3 | -3.83 | 39.30 | 0.9997 | 82.4 |
| # 4 | -3.78 | 38.88 | 0.9994 | 83.9 |
| # 5 | -3.72 | 38.79 | 0.9995 | 85.7 |
| # 6 | -3.62 | 38.62 | 0.9989 | 88.9 |
| # 7 | -3.78 | 39.11 | 0.9998 | 83.9 |
| # 8 | -3.73 | 38.98 | 0.9975 | 85.4 |
| # 9 | -3.77 | 39.32 | 0.9989 | 84.2 |
| # 10 | -3.76 | 38.40 | 0.9997 | 84.5 |
| # 11 | -3.77 | 38.49 | 0.9988 | 84.2 |
| # 12 | -3.74 | 38.40 | 1.0000 | 85.1 |
| Average | -3.75 | 38.9 | 0.9992 | 84.8 |
| Minimum | -3.83 | 38.4 | 0.9975 | 82.4 |
| Maximum | -3.62 | 39.3 | 1 | 88.9 |

**Table S2.** Measured eDNA concentration of each life history stage of artificially reared Japanese eels. 500 mL of seawater was collected from each outlet of three 30 L seawater tanks (Tank1, 2 and 3), each containing a single individual stage. One 500 mL seawater sample was analyzed in three replicates (Replicate 1, 2 and 3). ND shows that eDNA is not detected.

| Life history stage | Measured eDNA concentration (copies µL^-1^) | | | | | | | | | | |
| --- | --- | --- | --- | --- | --- | --- | --- | --- | --- | --- | --- |
|  | Tank 1 | | |  | Tank 2 | | |  | Tank 3 | | |
|  | Replicate 1 | Replicate 2 | Replicate 3 |  | Replicate 1 | Replicate 2 | Replicate 3 |  | Replicate 1 | Replicate 2 | Replicate 3 |
| Egg | ND | ND | ND |  | ND | ND | ND |  | ND | ND | ND |
| Preleptocephalus | ND | ND | ND |  | ND | ND | 0.2 |  | ND | ND | 0.1 |
| Leptocephalus | 0.6 | 1.0 | 2.6 |  | 1.5 | 4.0 | 3.1 |  | 3.1 | 3.1 | 0.9 |
| Glass eel | 0.2 | 0.5 | 0.4 |  | 0.6 | 0.2 | 0.1 |  | 0.6 | 0.4 | 0.5 |
| Elver | 9.3 | 9.6 | 9.4 |  | 9.0 | 7.8 | 5.9 |  | 4.6 | 9.1 | 4.8 |
| Yellow eel | 8.4 | 5.2 | 6.5 |  | 115.4 | 156.0 | 128.0 |  | 179.6 | 164.2 | 210.6 |
| Silver eel | 336.0 | 333.2 | 211.9 |  | 206.6 | 206.5 | 205.8 |  | 467.2 | 410.1 | 463.4 |
